# From Gut to Heart: Targeting Trimethylamine N-Oxide as a Novel Strategy in Heart Failure Management

**DOI:** 10.3390/biom15101447

**Published:** 2025-10-13

**Authors:** Zehui Ding, Yunfeng Yu, Jiaming Wei, Ziyan Wang, Ruifang Lin, Ya Li, Zhihua Guo

**Affiliations:** 1 First Clinical College of Chinese Medicine, Hunan University of Chinese Medicine, Changsha 410208, China; dingzehui@stu.hnucm.edu.cn (Z.D.); yuyunfeng@stu.hnucm.edu.cn (Y.Y.); 20212011@stu.hnucm.edu.cn (Z.W.); 2 College of Chinese Medicine, Hunan University of Chinese Medicine, Changsha 410208, China; 004916@hnucm.edu.cn (J.W.); 20232073@stu.hnucm.edu.cn (R.L.); 3 College of Pharmacy, Hunan University of Chinese Medicine, Changsha 410208, China; 003872@hnucm.edu.cn; 4 Key Laboratory of Chinese Medicine Intelligent Diagnosis and Treatment of Chronic Diseases in General Universities of Hunan Province, Changsha 410208, China

**Keywords:** trimethylamine N-oxide (TMAO), heart failure, gut microbiota, treatment, diet regulation, probiotics, inhibitors, flavin-containing monooxygenases (FMOs)

## Abstract

Heart failure (HF) marks the culmination of numerous cardiac pathologies, presenting a major medical hurdle in prevention and treatment. In recent years, with the advancements in genomics and metabolomics, research has demonstrated that gut microbiota plays a significant role in the pathogenesis of HF. Trimethylamine N-oxide (TMAO) is a gut microbiota-derived metabolite and primarily sourced from foods abundant in choline, L-carnitine, and betaine. Research has shown that patients with HF exhibit higher levels of TMAO. Accumulating evidence has indicated that TMAO directly or indirectly mediates the occurrence and development of HF through multiple mechanisms. Furthermore, TMAO functions as a crucial prognostic marker in HF. Therefore, TMAO emerges as a potential therapeutic target for HF. This article reviews the generation and metabolic pathways of TMAO, emphasizes its pathophysiological mechanisms in HF, and explores promising therapeutic approaches targeting TMAO, offering novel insights and strategies for HF management.

## 1. Introduction

Heart failure (HF) represents a complex clinical syndrome, marking the culmination of diverse cardiac pathologies. It is characterized by symptoms and signs arising from structural or functional impairments in ventricular filling or ejection, accompanied by elevated natriuretic peptide levels or objective evidence of pulmonary or systemic congestion [1,2]. Globally, HF affects more than 56 million individuals, constituting over 1% of the world’s population [3,4]. As populations age and cardiovascular disease (CVD) rates rise, the prevalence and mortality of HF continue to increase [2,3]. The primary etiologies include ischemic heart disease (IHD), rheumatic and valvular heart disease, cardiomyopathies, amyloidosis, certain infectious diseases, and endocrine disorders [3]. Based on left ventricular ejection fraction (LVEF), HF is classified into three subtypes: HF with reduced ejection fraction (HFrpEF, LVEF < 40%), HF with mid-range ejection fraction (40% ≤ LVEF < 50%), and HF with preserved ejection fraction (HFpEF, LVEF ≥ 50%) [2,5]. Multiple mechanisms—such as inflammation, oxidative stress, disrupted energy metabolism, and pathological cardiac remodeling—can initiate or worsen HF. Although HF prediction and diagnosis are relatively straightforward, effective prevention and treatment still pose significant challenges [6]. Despite advances in pharmacotherapy and interventions, HF persists as a leading cause of mortality worldwide, underscoring the need for deeper insights into its pathophysiology and innovative therapeutic targets. Recent research has highlighted the role of the gut–heart axis in HF, proposing that gut microbiota dysbiosis may aggravate myocardial remodeling and cardiac dysfunction. This occurs through metabolic disturbances, immune-mediated systemic inflammation, and intestinal barrier damage, leading to translocation of gut-derived toxins [7,8,9,10]. Hence, selective modulation of the gut microbiome emerges as a possible breakthrough in addressing HF.

The gut microbiota can be conceived of as a massive virtual metabolic organ within the human body, encompassing more than 30 trillion microorganisms [11]. Its composition is influenced by host age, sex, diet, antibiotic use, environmental factors, and genetics, among other variables. A balanced gut microbiota is essential for cardiovascular homeostasis, and its disruption could induce atherosclerosis (AS), arrhythmias, and other CVDs [8]. The involvement of gut microbiota in HF has gained increasing attention, with evidence indicating that alterations in microbial communities and their metabolites play a pivotal part in HF occurrence and progression [12,13]. Gut microbiota dysbiosis may worsen HF by impairing metabolic, immune, and barrier functions [14]. Gut microbial metabolites, such as short-chain fatty acids (SCFAs), trimethylamine N-oxide (TMAO), bile acids (BAs), and lipopolysaccharide (LPS; an intrinsic component of the Gram-negative bacterial cell wall that exerts its toxicity through lipid A and is released as endotoxin upon bacterial death and lysis), also exert either beneficial or detrimental effects in HF [15]. For example, SCFAs are generally regarded as advantageous, whereas TMAO and LPS may intensify HF [9].

Further studies reveal the gut microbiota-derived metabolite TMAO as a key mediator connecting HF with intestinal homeostasis, potentially bridging microbial dysbiosis and cardiac pathophysiology. HF patients exhibit elevated TMAO levels [16,17], which may result from intestinal and renal alterations associated with the disease. The gut microbiota composition in HF patients differs significantly from that in healthy individuals, typically showing an increased abundance or proportion of *Firmicutes* (e.g., *Ruminococcus gnavus* [18]) and *Proteobacteria* (e.g., *Escherichia*, *Klebsiella*, *Campylobacter*, and *Shigella* [19,20]) [21]. These shifts likely promote the production of TMAO and its precursor trimethylamine (TMA). Moreover, HF often coincides with elevated intestinal permeability and impaired renal function, which facilitate TMAO entry into circulation and diminish its excretion. TMAO has also been shown to predict the onset and progression of HF in certain instances [22] and serves as a valuable prognostic marker [21,23,24]. Increasing evidence indicates that TMAO plays an important regulatory role in HF [25,26,27,28].

Although several studies have offered preliminary insights into how TMAO influences HF and into potential TMAO-targeted therapies, direct evidence of causal involvement remains limited, and comprehensive mechanistic and therapeutic investigations are still needed. Accordingly, this review examines the role of TMAO in HF and focuses on potential therapeutic strategies targeting TMAO, thereby offering new targets and perspectives for HF management.

## 2. The Generation and Metabolism of TMAO

**Figure 1:**
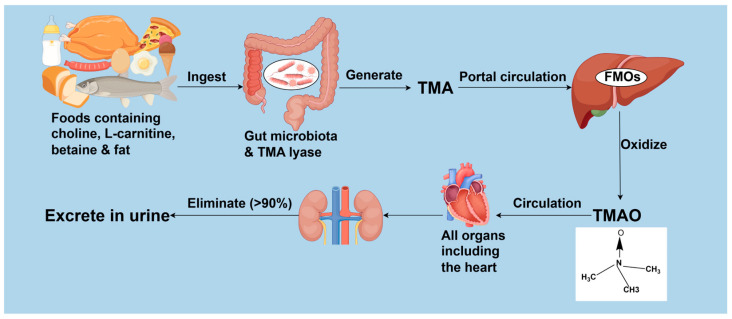
The generation and metabolism of TMAO. TMA: trimethylamine; FMOs: flavin-containing monooxygenases; TMAO: trimethylamine N-oxide. Created by Fidraw (www.figdraw.com, accessed on 6 August 2025).

TMAO, a compound bearing the molecular formula (CH_3_)_3_NO [29], stands as a gut microbiota-derived metabolite increasingly recognized as a vital contributor to intestinal-origin CVDs [7]. Gut microbiota metabolizes choline, L-carnitine, betaine, and other choline- or TMA-rich compounds into TMA, a transformation catalyzed by specific enzymes notably including choline TMA-lyase CutC and its activator CutD, carnitine monooxygenase CntA/B, betaine reductase, TMAO reductase, and the CntA/B homologs YeaW/X [30,31,32,33]. The resulting TMA permeates the intestinal barrier, enters circulation, and subsequently travels via the portal circulation to the liver, where it is rapidly oxidized by hepatic flavin-containing monooxygenases (FMOs) into TMAO [34]. TMAO then circulates systemically [35]. In healthy individuals, over 90% of TMAO is eliminated by the kidneys and rapidly excreted in urine [36]. The generation and metabolic process of TMAO is detailed in Figure 1.

Systemic TMAO concentrations are influenced by multiple factors, primarily determined by three key processes: intestinal generation, hepatic conversion, and renal elimination. Dysregulation in any of these processes may elevate TMAO levels. Dietary choline is obtained mainly from meat, eggs, dairy products, fish, grains, and foods derived from them [37]. The primary dietary source of L-carnitine is red meat [38]. Betaine occurs naturally in foods like beets, spinach, grains, and shrimp [39]. Gut microbiota metabolize these dietary precursors into TMA via specific key enzymes. However, the gene clusters encoding these enzymes occur only in certain intestinal bacteria [30]. These clusters are more prevalent among the gut bacteria of carnivores and omnivores than herbivores [30], initiating increased TMAO generation [31]. High-fat diets (HFDs) have also been found to elevate TMAO concentrations [32]. Accordingly, TMAO synthesis is influenced by the composition and function of gut microbiota as well as dietary intake. Concurrently, FMO activity affects TMAO generation; for instance, liver diseases such as non-alcoholic fatty liver disease can upregulate FMO expression [40], promoting TMA oxidation. Among various FMOs that convert TMA to TMAO, FMO3 exhibits the highest activity [41]. Knocking out or silencing of hepatic FMO3 in mice decreases circulating TMAO levels [36,42]. Renal insufficiency may also elevate TMAO due to reduced excretion [43]. A limited number of studies suggested that dietary modulation of TMAO levels may not occur through direct regulation of FMO3 activity [44,45]. However, direct evidence remains scarce that elevated TMAO arises solely from increased TMA formation or impaired renal excretion, independent of FMO-mediated oxidation. Consequently, high TMAO levels likely reflect the combined influence of increased TMA precursors, enhanced FMO conversion efficiency, and impaired renal excretion—three dynamically interacting factors that should be considered together.

Moreover, TMAO concentrations correlate positively with age [46,47]. Genetic factors also influence TMAO production and metabolism. For example, mutations in the FMO3 gene can reduce or abolish enzymatic activity, impairing TMA-to-TMAO conversion and leading to toxic TMA accumulation. This condition, trimethylaminuria (TMAU), is characterized by a fish-like odor in bodily secretions [48] and represents the most direct genetic influence on TMAO metabolism. Host genetics also partially shape gut microbiota composition and function. Individual genetic backgrounds may indirectly modulate TMA production by influencing microbial community structure, thereby contributing to interindividual variation in TMAO levels. A large-scale population study linked variability in TMAO and its precursors to the abundance of *Ruminococcaceae* genera and the *CutC* gene in gut microbiota [33]. Certain TMA-producing bacteria, including some *Firmicutes*, show heritability [49,50]. Additionally, circulating TMAO concentrations vary substantially among healthy adults across geographic regions. In the United States, the median circulating TMAO level in healthy populations was 3.5 μmol/L [51,52], whereas Fang et al. [53] recently documented a lower median of 1.70 μmol/L among healthy Chinese adults. These disparities may arise from genetic, dietary, and ethnic factors.

In conclusion, TMAO levels correlate with various factors, including dietary patterns, gut microbiota structure, activity of TMAO-producing enzymes such as TMA lyase and FMOs, renal function, age, genetics, ethnicity, and geographical region.

## 3. The Mechanisms by Which TMAO Regulates HF

### 3.1. Inflammation

Inflammation is closely linked to HF [14]. Higher concentrations of TMAO, such as 600 μmol/L, have been shown to enhance the expression of inflammatory genes and activate proinflammatory cytokines, thus amplifying inflammatory responses [54]. Boini et al. [55] experimentally demonstrated that TMAO stimulated the secretion of inflammatory cytokines, including interleukin (IL)-1β, by activating the nucleotide-binding oligomerization domain-like receptor containing pyrin domain 3 (NLRP3) inflammasome, leading to endothelial inflammation. TMAO also inhibited the deacetylation of superoxide dismutase 2 (SOD2) by suppressing Sirtuin 3 (SIRT3), thereby reducing SOD2 activity. This process promoted mitochondrial reactive oxygen species (mtROS) generation, leading to elevated overall reactive oxygen species (ROS) levels [56,57]. This increased thioredoxin-interacting protein (TXNIP) levels, subsequently activating the NLRP3 inflammasome [57]. Such activation elicited a dose- and time-dependent release of IL-1β and IL-18 [58], culminating in an inflammatory response.

Moreover, TMAO may influence HF by modulating inflammatory pathways. Zhang and colleagues [59] reported that TMAO could possibly exacerbate myocardial inflammation in mice by elevating TNF (tumor necrosis factor)-α and reducing IL-10 levels. Additionally, their study showed that TMAO supplementation (120 mg/kg) in drinking water may counteract the cardioprotective benefits of voluntary exercise [59].

### 3.2. Abnormal Energy Metabolism

Abnormal energy metabolism plays a pivotal role in the development of HF. In HF, due to the increased dependence of cardiac metabolism on glucose, the primary energy source shifts from fatty acid (FA) oxidation to glucose metabolism [60]. Additionally, hypoxia reduces oxidative phosphorylation (OXPHOS) and enhances glycolysis [61]. These metabolic alterations contribute to an insufficient energy supply, thus worsening the condition of HF.

Elevated TMAO levels are strongly linked to impaired cardiac energy metabolism. HFD-induced TMAO accumulation inhibited mitochondrial activity and consequently compromised energy metabolism [62]. Saaoud et al. [54] revealed that 600 μmol/L TMAO initiated a metabolic switch from OXPHOS to glycolysis. This shift occurred by upregulating mitochondrial regulators such as perilipin 4 (PLIN4), overlapping activity with m-AAA protease 1 (OMA1), and oxoglutarate dehydrogenase L (OGDHL), ultimately disrupting energy homeostasis. Brown adipose tissue (BAT) enhances thermogenesis and supports cardiac function [63,64], but hypoxia induces brown adipocyte apoptosis, resulting in BAT dysfunction [28]. In physiological conditions, BAT absorbs and metabolizes circulating choline, potentially to maintain its cellular membrane integrity. In a murine model of HF, BAT dysfunction led to choline accumulation, which may be partially converted to TMAO via the choline-TMA-TMAO pathway; this increased TMAO synthesis exacerbated HF [28]. This study further revealed that TMAO significantly attenuated creatine phosphate (CP) and adenosine triphosphate (ATP) concentrations in cardiac tissue by suppressing mitochondrial complex IV activity, further impairing cardiac energy metabolism. This led to a vicious cycle of BAT dysfunction and cardiac dysfunction ultimately [28].

In addition, impaired calcium ion (Ca^2+^) handling in myocardial cells increases the energy demand of the failing heart [65], compromising both systolic and diastolic function and worsening HF. TMAO disrupts Ca^2+^ regulation in cardiac cells. In endothelial cells, 100 μmol/L TMAO suppressed purinergic response-induced increase in Ca^2+^ influx and prolongation of Ca^2+^ signaling, indicating loss of Ca^2+^ homeostasis [66]. High glucose and high fat could also induce TMAO production, possibly predisposing individuals to Ca^2+^ overload [67]. Esposito and coworkers [68] proved that 100 μmol/L TMAO transiently suppressed contractile recovery of myocardial cells by elevating intracellular Ca^2+^ currents.

### 3.3. Oxidative Stress

Oxidative stress contributes to the pathophysiological HF via multiple mechanisms. Studies have demonstrated that TMAO can mediate oxidative stress. TMAO concentrations were remarkably higher in preeclampsia patients. Transplanting fecal microbiota from these patients into antibiotic-treated mice resulted in pronounced oxidative stress in the recipients [69]. In addition, TMAO can provoke oxidative stress by distinct pathways. A study involving mice and healthy human subjects revealed that TMAO promoted oxidative stress by increasing superoxide levels and impairing endothelial nitric oxide synthase (eNOS) activity [46]. Chen et al. [57] confirmed that TMAO, at concentrations of 150, 300, 600, and 900 μmol/L, augmented ROS generation, particularly mtROS, through modulating the SIRT3-SOD2-mtROS signaling cascade, thus fostering oxidative stress. Moreover, TMAO induced oxidative stress through activation of NADPH oxidase 2 (NOX2) [70].

Elevated TMAO levels have been demonstrated to initiate HF through oxidative stress. Recent research revealed that TMAO induced oxidative stress by downregulating piezo type mechanosensitive ion channel component 1 (PIEZO1), thereby exacerbating cardiac diastolic dysfunction in mice suffering from HFpEF [25]. Therefore, decreasing TMAO concentrations and alleviating oxidative stress appear to be a viable therapeutic approach for HF.

### 3.4. Myocardial Remodeling

Myocardial remodeling stands as a significant mechanism underlying the occurrence and progression of HF. Its primary features consist of pathological cardiomyocyte hypertrophy and myocardial fibrosis. TMAO directly influences this process. Research in mice indicated that a Western diet (WD) or TMAO supplementation increased TMAO levels, promoted myocardial fibrosis, and impaired cardiac function [59]. Studies demonstrated that TMAO stimulated myocardial remodeling in Sprague-Dawley rats and triggered hypertrophic responses in rat cardiomyocytes, enlarging cell size while upregulating hypertrophy markers such as atrial natriuretic peptide (ANP) and β-myosin heavy chain (β-MHC). This effect might involve activation of the transforming growth factor (TGF)-β1/Smad3 signaling pathway [71]. Another study indicated that transverse aortic constriction-induced HF in mice correlated with elevated TMAO levels, exacerbated myocardial hypertrophy and fibrosis, and compromised cardiac function. The mechanisms could be associated with activation of the TGF-β1/Smad3 and p65 nuclear factor (NF)-κB signaling pathways [27].

TMAO aggravates HF by affecting adverse cardiac remodeling. Organ et al. [72] discovered that diets containing choline (1.2%) or TMAO (0.12%) markedly increased circulating TMAO and brain natriuretic peptide (BNP) levels in HF mice. Concurrently, left ventricular (LV) fractional shortening and ejection fraction were decreased, accompanied by exacerbated LV remodeling, intensified myocardial fibrosis, and deteriorated cardiac dysfunction, ultimately accelerating HF progression. Subsequent experiments showed that discontinuing TMAO intake or inhibiting TMA lyase effectively lowered the HF marker BNP levels, suppressed numerous profibrotic gene expressions, and ultimately mitigated ventricular remodeling and cardiac dysfunction [73].

### 3.5. Indirect Mechanisms

TMAO indirectly contributes to the emergence and progression of HF by mediating several mechanisms such as endothelial dysfunction [46], renal insufficiency [74], elevated blood pressure [75], enhanced platelet activity and thrombosis [34], and lipid metabolism disorders [9,31].

#### 3.5.1. Endothelial Dysfunction

Endothelial dysfunction plays a crucial part in AS-related CVDs and ensuing HF. Studies uncovered a distinct association between TMAO and endothelial dysfunction biomarkers in patients suffering from HF [9]. Elevated serum TMAO levels disrupt vascular endothelial integrity and exacerbate endothelial dysfunction, promoting vascular dysfunction, inflammation, fibrosis, and AS [9], ultimately increasing CVD risk [9,54].

Multiple studies have explored the mechanisms by which TMAO causes endothelial dysfunction. In both aged mice and middle-aged or elderly human subjects, TMAO levels and the severity of endothelial dysfunction increased significantly with age [46]. Additionally, TMAO exacerbated endothelial dysfunction by promoting inflammation and oxidative stress, suppressing eNOS activity and impairing NO (nitric oxide)-mediated vasodilation. These findings suggested that elevated TMAO levels during aging likely reduce eNOS-derived NO bioavailability through vascular inflammation and oxidative stress, contributing to age-related endothelial dysfunction [46]. Additional research revealed that TMAO induced endothelial dysfunction via the ROS-TXNIP-NLRP3 inflammasome signaling pathway [27]. Evidence indicated that TMAO fostered endothelial dysfunction and vascular calcification through the activation of the NLRP3 inflammasome and NF-κB signaling [76]. Kim et al. [77] revealed that TMAO (50 and 100 μmol/L) mediated chromatin remodeling in coronary artery endothelial cells by augmenting histone methylation, fostering endothelial-to-myofibroblast transformation and vascular fibrosis, thus inducing ischemic HF [78].

#### 3.5.2. Renal Insufficiency

Studies have shown that patients with deficient renal function have higher blood TMAO levels [9,43], and a notable inverse relationship exists between TMAO levels and estimated glomerular filtration rate [79]. Rising TMAO concentrations may worsen renal insufficiency and raise all-cause mortality in chronic kidney disease patients through inducing progressive renal fibrosis and dysfunction [43].

In HF, neurohormonal activation ultimately results in hypoperfusion and dysfunction in multiple organs, the kidneys among them. Likewise, the significance of defective renal function in the advancement of HF is not negligible. TMAO is linked to the onset and development of both HF and renal insufficiency [8]. On one hand, TMAO promotes water and sodium retention by triggering glomerular interstitial fibrosis and renal dysfunction, indirectly deteriorating HF [43,80]. On the other hand, chronic heart failure (CHF) frequently contributes to progressive renal function impairment [74], which decreases TMAO excretion and increases circulating TMAO concentrations. These elevated TMAO levels further exacerbate HF progression. For instance, in a rat model of cardiorenal syndrome featuring both HF and renal insufficiency, increased blood TMAO levels exacerbated cardiac and renal dysfunction [74].

#### 3.5.3. Elevated Blood Pressure

Elevated blood pressure is a primary inducer of HF. Uncontrolled chronic hypertension (HTN) could induce LV remodeling via biochemical and neurohumoral mechanisms, which may subsequently result in hypertensive heart disease and, in its most severe form, progress to HF.

TMAO is closely associated with enhanced blood pressure. A systematic review and meta-analysis revealed an obvious positive dose-dependent correlation between circulating TMAO levels and HTN prevalence [81]. Jiang et al. [75] reported that 1% TMAO enhanced angiotensin (ANG) II-induced vasoconstriction and acute pressor responses by activating protein kinase R-like endoplasmic reticulum kinase-associated pathways, consequently worsening HTN. Another research indicated that higher TMAO concentrations (30 μmol/L) increased systolic blood pressure in both mice and human subjects by inducing advanced glycation end-product accumulation and oxidative stress [82]. These observations suggest that TMAO could function as a potential biomarker for HTN.

#### 3.5.4. Increased Platelet Activity and Thrombosis

Excessive platelet activation may facilitate thrombosis. Research has shown that elevated platelet activity and thrombosis play vital roles in the onset and advancement of HF [83]. Plasma TMAO might trigger excessive platelet activation and thrombosis in humans [42,84]. Clinical studies further indicated that TMAO predicted the likelihood of thrombotic events, like myocardial infarction (MI) and stroke [34,85]. In mice, plasma TMAO concentrations correlated positively with thrombosis rates, and dietary supplementation with choline or TMAO increased platelet activity and accelerated thrombosis [34,84,85]. Similarly, in humans, choline intake resulted in elevated TMAO levels, which enhanced platelet reactivity, ultimately facilitating thrombosis [86]. Zhu and colleagues [34] confirmed that 100 μmol/L TMAO potentiated platelet hyperreactivity by stimulating intracellular Ca^2+^ release, thereby raising thrombosis risk.

Additionally, TMAO can contribute to CVDs via increased platelet activity and thrombosis [87]. Hence, attenuating TMAO production may serve as a therapeutic target to curb excessive platelet activation and thrombosis, improving clinical outcomes in CVDs, including HF.

#### 3.5.5. Abnormal Lipid Metabolism

Abnormal lipid metabolism emerges as a key pathogenic factor in HF, and correcting lipid metabolism is crucial for its treatment [88]. TMAO accumulation has been confirmed to cause abnormal lipid metabolism [9,89], consequently increasing the risk of diverse CVDs, HF among them. Elevated TMAO disrupts lipid metabolism through mechanisms such as inhibiting reverse cholesterol transport (RCT), promoting macrophage foam-cell formation, and modifying BA synthesis.

First, TMAO suppresses RCT and subsequently disrupts cholesterol metabolism. TMAO and its precursors, choline and carnitine, impede RCT in the body through a gut microbiota-dependent mechanism [31]. Second, TMAO facilitates cholesterol accumulation in macrophages and the formation of foam cells [9,90]. Wang et al. [91] discovered that TMAO enhanced phagocytic activity and cholesterol content in peritoneal macrophages by upregulating scavenger receptor A and CD36, thereby promoting foam-cell formation. Third, TMAO also restricts the production of BA. In apolipoprotein E knockout (ApoE^−/−^) mice fed a diet containing 0.3% TMAO, TMAO altered the BA profile and suppressed hepatic BA synthesis by specifically inhibiting the classical pathway, leading to abnormal lipid metabolism and remarkably increased blood lipid concentrations [89]. Also, according to He and coworkers [92], TMAO increased hepatic cholesterol accumulation by reducing fecal excretion of acidic sterols, contributing to hyperlipidemia.

**Figure 2:**
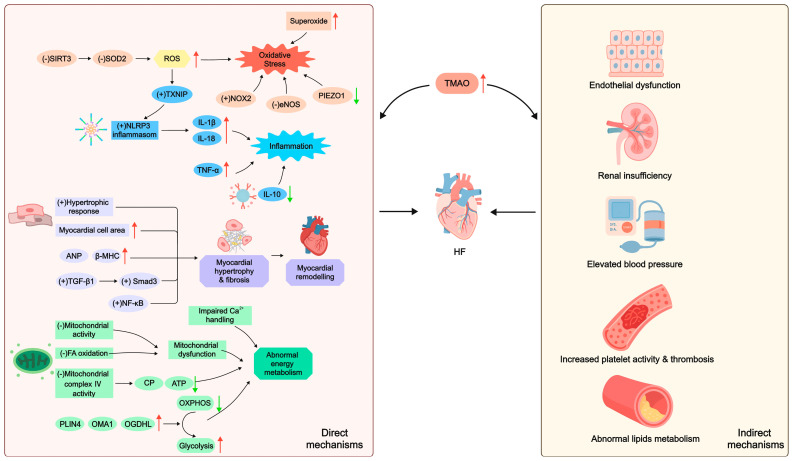
The mechanisms by which TMAO regulates HF. An upward arrow (↑) indicates an increase. A downward arrow (↓) indicates a decrease. A plus sign (+) indicates promotion. A minus sign (−) indicates inhibition. SIRT3: Sirtuin 3; SOD2: superoxide dismutase 2; ROS: reactive oxygen species; TXNIP: thioredoxin-interacting protein; NOX2: NADPH oxidase 2; eNOS: endothelial nitric oxide synthase; PIEZO1: piezo type mechanosensitive ion channel component 1; NLRP3: nucleotide-binding oligomerization domain-like receptor containing pyrin domain 3; IL: interleukin; TNF: tumor necrosis factor; ANP: atrial natriuretic peptide; β-MHC: β-myosin heavy chain; TGF: transforming growth factor; NF: nuclear factor; Ca^2+^: calcium ion; FA: fatty acid; CP: creatine phosphate; ATP: adenosine triphosphate; PLIN4: perilipin 4; OMA1: overlapping activity with m-AAA protease 1; OGDHL: oxoglutarate dehydrogenase L; OXPHOS: oxidative phosphorylation; TMAO: trimethylamine N-oxide; HF: heart failure.

In summary, TMAO directly or indirectly modulates HF through multiple pathophysiological mechanisms, including inflammation, abnormal energy metabolism, oxidative stress, and myocardial remodeling [25,28,59,72] (Figure 2). Moreover, these pathways often interact, creating a pernicious cycle that accelerates the advancement of HF even more [93].

## 4. Potential Therapeutic Approaches

Multiple studies have demonstrated that elevated TMAO levels are independently associated with an increased risk of adverse outcomes in patients with HFpEF [22,94,95] and HFrEF [94,96,97], suggesting its potential as a prognostic biomarker. Targeting TMAO has therefore emerged as a viable therapeutic approach. In HFpEF, strategies to lower TMAO—such as dietary modification, physical activities, inhibitors, or medications like sodium-glucose cotransporter-2 (SGLT2) inhibitors—may reduce systemic inflammation, improve metabolism, and mitigate myocardial remodeling. For HFrEF, interventions including aspirin, angiotensin-converting enzyme inhibitors (ACEIs), or statins can lower TMAO, which may help alleviate vascular inflammation and atherosclerosis, improve myocardial ischemia, and lead to better clinical outcomes.

### 4.1. Diet Regulation

Current evidence indicates that dietary intervention could effectively lessen circulating TMAO levels, probably aiding in the prevention and treatment of HF [21]. As noted, consuming foods abundant in TMA precursors—including choline, L-carnitine, and betaine—or high-fat foods increases TMAO concentrations in humans. In animal studies, a choline-rich diet significantly increased TMAO and BNP levels, worsened ventricular remodeling, impaired cardiac function, and further aggravated HF [72]. Mo et al. [32] demonstrated that an HFD elevated plasma TMAO concentrations in naturally aging rats. In healthy rats, prolonged HFD intake triggered low-grade systemic inflammation and diminished beneficial SCFA levels, while long-term fructose consumption heightened oxidative stress, elevated blood pressure, and expanded *Enterobacter* and *Escherichia coli* populations [98]. The WD, distinguished by high amounts of TMA precursors, fats, and sugars [9,47,62], is strongly associated with increased TMAO concentrations [52]. Consuming WD alters gut microbiota composition, diminishes its diversity, and consequently promotes TMAO production, rising blood TMAO concentrations, even in healthy people [99]. The WD has been proven to elevate circulating TMAO levels, thus inducing cardiac fibrosis and systemic inflammation. These pathological processes can subsequently cause cardiac dysfunction, heightening the risk of developing AS, coronary artery diseases (CADs), and HF [9,21,47,62]. Furthermore, animal experiments demonstrated that a high-salt diet may reduce the ratio of SCFAs to TMAO, lower *Lactobacillus* abundance, and exacerbate immune and inflammatory responses [100]. Additionally, insufficient dietary fiber could worsen gut microbiota dysbiosis [101]. Hence, it is advisable to steer clear of overindulging in foods rich in TMA precursors, fats, sugars, or salts [99], while augmenting dietary fiber intake.

Studies have shown that consuming red meat raises TMAO concentrations. Decreasing consumption of choline- or L-carnitine-rich foods such as red meat can hinder TMAO synthesis, ultimately reducing HF incidence [7,52]. A plant-based diet primarily consists of fruits, vegetables, seeds, nuts, legumes, whole grains, and herbal plants. It may adhere strictly to a vegetarian diet or incorporate moderate quantities of animal-derived foods like fish, seafood, dairy products, and eggs [102]. This dietary pattern has been demonstrated to modulate gut microbiota, augment the generation of advantageous metabolites such as SCFAs, and diminish TMAO levels [103,104,105]. The Mediterranean diet (MD), a well-established healthy pattern, emphasizes ample fruits, vegetables, nuts, and whole grains, along with moderate meat, eggs, sugar, olive oil, and wine [93]. It is low in saturated FAs, salt, and phosphates, but rich in unsaturated FAs, antioxidants (such as polyphenols, vitamins, and flavonoids), nitrates, and dietary fiber [9,37,67]. Healthy diets like the MD could modulate gut microbiota composition and its metabolites, influencing the occurrence and progression of HF. Studies have indicated that MD mitigates oxidative stress and inflammation while enhancing antioxidant capacity and endothelial function by augmenting the gut microbial diversity and abundance, raising SCFA production, and reducing TMAO concentrations. These effects collectively improve cardiac performance and lower CVD risk [9,106,107]. Adherence to the MD has been linked to decreased HF incidence and mortality [108], and it has exhibited additional benefits such as restoring gut microbial balance and lessening TMAO concentrations [109]. Estruch and colleagues [110] demonstrated that MD consumption, especially when enriched with extra-virgin olive oil, effectively attenuated TMAO levels, thus diminishing major cardiovascular events such as MI and stroke. Kaluza et al. [111] uncovered that certain foods, including vegetables, fruits, and nuts, lessened HF risk in individuals with smoking addictions by modifying gut microbiota, suppressing oxidative stress, and inhibiting cell death. Merques and coworkers [112] revealed that a high-fiber diet regulated gut microbiota, lowered blood pressure, and ameliorated cardiac fibrosis and LV hypertrophy, thereby contributing to the prevention of HF. Furthermore, obese patients following a low-calorie diet may experience decreased TMAO concentrations [113,114]. In summary, healthy eating serves as a practical and economical way to manage HF, mainly by modulating gut microbiota and their metabolites [93].

### 4.2. Physical Activities

Tailored exercise training can enhance exercise capacity and quality of life in patients with HF, improve survival rates, and lower the likelihood of HF-related hospitalization [115,116]. Additionally, physical activities have been shown to modulate gut microbiota [117], leading to lower TMAO levels. For example, sprint training decreased serum and urinary TMAO concentrations in male participants [118,119]. Physical activity also notably reduced plasma TMAO levels in a study of 16 obese adults [113]. Zhang et al. [120] discovered that voluntary wheel running in mice alleviated gut microbiota dysbiosis and decreased serum levels of TMAO and its precursors, TMA and betaine.

Physical activities confer beneficial effects on the heart by downregulating TMAO concentrations. After being fed a WD, mice that engaged in voluntary exercise demonstrated reduced plasma TMAO levels, diminished myocardial inflammation and fibrosis, and enhanced cardiac function compared with sedentary controls [59]. Brandao and colleagues [121] found that a combined exercise regimen—incorporating both strength and aerobic training—decreased TMAO levels, thereby mitigating cardiovascular risk and strengthening physical function in obese women.

### 4.3. Probiotics, Prebiotics, and Synbiotics

Probiotics, prebiotics, and synbiotics serve as prevalent therapeutic avenues for preserving gut microbiota homeostasis [122]. Probiotics, live microorganisms capable of adjusting the quantity and composition of gut microbiota, confer health benefits [35]. Prebiotics consist of non-digestible substrates selectively utilized by beneficial microorganisms in the host to promote their growth and/or activity, ultimately improving host health [122,123]. Synbiotics combine live microorganisms with substrates selectively metabolized by host microbiota, effectively integrating the benefits of both probiotics and prebiotics to foster host well-being. The synergistic and complementary effects of live microorganisms and substrates allow synbiotics to effectually influence microbiota composition and immune function [124].

Research has shown that probiotics, prebiotics, or synbiotics can remodel the gut microbiota in animals, thereby reducing gut-derived TMA synthesis, inhibiting or blocking the TMA/TMAO pathway, and ultimately decreasing TMAO levels [7]. Probiotics, including *Lactobacillus plantarum* ZDY04, *Bifidobacterium breve* Bb4, *Bifidobacterium longum* BL1 and BL7, and *Lacticaseibacillus rhamnosus* L34, may decrease TMAO concentrations by adjusting gut microbiota and restricting TMA generation [44,125,126]. Wang et al. [125] discovered that *Bifidobacterium* lessened the abundance of TMA-producing *Ruminococcaceae* UCG-009 and UCG-010, thereby lowering plasma TMAO concentrations. Among *lactobacilli* strains with robust adherence capability, *Lactobacillus amylovorus* LAM1345 and *Lactiplantibacillus plantarum* LP1145 individually reduced serum TMAO in choline-fed mice. Whereas, a multistrain formula (MF) containing these two strains plus *Limosilactobacillus fermentum* LF33 exhibited the most pronounced reduction, likely due to the additive and synergistic actions in MF [127]. Furthermore, following a 12-week synbiotic intervention decreased serum TMAO and improved metabolic profiles in patients with dyslipidemia [128].

Probiotics, prebiotics, and synbiotics hold potential in treating CVDs, like HF, via various pathophysiological mechanisms [129,130]. The gut microbiota of HF patients showed reduced probiotic abundance and elevated harmful bacteria [131]. Several probiotics, such as *Lactobacillus plantarum* ZDY04 [44], *Lactobacillus rhamnosus* GG strain [132], and *Bifidobacterium lactis* Probio-M8 [133], reduced TMAO concentrations, aid in CVD management, and diminish adverse cardiovascular events, indicating TMAO inhibition contributed to their cardioprotective effects. Sánchez-Quintero et al. [134] transplanted gut microbiota from patients with IHD into mice, elevating TMAO concentrations in recipients. Prebiotic essential oils from parsley (*Petroselinum crispum*) and rosemary (*Rosmarinus officinalis*) lessened plasma TMAO levels by reshaping gut microbiota in these mice. Similarly, thyme (*Thymus vulgaris*) and oregano (*Origanum vulgare*) essential oils functioned as effective prebiotics and produced comparable effects in gnotobiotic mice colonized with gut microbiota from CAD patients [135]. Synbiotics also reduced NT-proBNP levels and ameliorated the inflammatory status in patients with CHF [130]. As a result, probiotics, prebiotics, synbiotics, and their derivatives may offer safe and effective strategies for HF prevention and treatment through mechanisms including modulating gut microbiota, reducing TMAO levels, and maintaining host intestinal homeostasis [93].

Archaea, ancient single-celled prokaryotes inhabiting extreme environments and morphologically similar to bacteria [136], are also present in the human gastrointestinal tract. Certain archaea partially convert gut-derived TMA into methane, reducing TMA content [137]. Ramezani and coworkers [138] found that intestinal colonization with *Methanobrevibacter smithii*, a methanogenic archae, reduced plasma TMAO in ApoE^−/−^ mice, consequently attenuating AS progression. Although current research on archaea-based therapies for CVDs is still limited, this approach appears highly promising.

### 4.4. Inhibitors

As previously mentioned, choline TMA lyase—which metabolizes choline into TMA—and FMOs—which oxidize TMA to produce TMAO—play critical parts in the TMAO synthesis and thus represent crucial targets for impeding TMAO production. Current methods for reducing TMAO generation typically focus on inhibiting TMA lyase or FMOs employing pharmacological inhibitors or genetic modification.

Choline TMA lyase (CutC), encoded by adjacent genes within a gene cluster, is a glycosyl radical enzyme. Both CutC and its activating protein CutD are critical for TMA biosynthesis in the gut microbiota [139]. 3,3-dimethyl-1-butanol (DMB), a choline analog, is the most widely studied inhibitor of this enzyme. It is naturally present in vinegar, extra virgin olive oil, wine, and grape seed oil; notably, extra virgin olive oil and wine are components of the MD [93,140]. Hence, DMB may partly account for the TMAO-lowering effects associated with the MD. Wang and colleagues [140] first showed in vitro and in vivo that DMB can partially inhibit TMA lyase activity in different microorganisms, blocking TMA formation from various substrates, including choline and carnitine, ultimately decreasing plasma TMAO concentrations. Additionally, DMB is non-toxic and non-lethal. Nevertheless, it does not inhibit TMA generation from certain substrates such as γ-butyrobetaine, nor does it affect TMA oxidation by FMO3 [140]. Based on DMB, the Hazen team developed a new generation of TMA lyase inhibitors: fluoromethylcholine (FMC) and iodomethylcholine (IMC). FMC and IMC irreversibly impede CutC/D, thereby effectively and continuously attenuating host TMA and TMAO levels. Furthermore, they exhibit poor absorption by the host, leave commensal bacteria unaffected, demonstrate limited systemic exposure, and are non-toxic and associated with a reduced risk of side effects [85]. Meanwhile, FMO inhibitors lower plasma TMAO by blocking hepatic TMA oxidation [141]. Methimazole, a high-affinity substrate of FMO3, acts as a competitive inhibitor, whereas indoles are non-substrate competitive FMO3 inhibitors. Both have been shown to suppress FMO3 activity and decrease TMAO concentrations [28,142]. However, the impact of methimazole on the thyroid cannot be overlooked. Therefore, inhibiting important TMAO-producing enzymes, specifically TMA lyase and FMO3, offers a plausible approach to lessening TMAO levels and ultimately managing CVDs like HF.

TMA lyase inhibitors have been extensively employed in animal models to hinder TMAO generation and ameliorate cardiovascular impairments. Studies have shown that DMB may attenuate inflammation, cardiomyocyte hypertrophy, and fibrosis, and improve cardiac function in mice with compromised hearts or HF, mainly through TMAO reduction. Furthermore, chronic DMB treatment exhibits no overt toxicity [25,27]. In mice fed WDs, DMB ameliorated excessive TMAO levels, vascular dysfunction, reduced exercise tolerance, and frailty. It also alleviated cardiac inflammation and fibrosis, consequently improving cardiac function and ultimately diminishing CVDs likelihood [143]. IMC, another TMA lyase inhibitor, reduced TMAO and BNP levels in HF mice, downregulated profibrotic gene expression, and mitigated TMAO-induced cardiac remodeling and dysfunction. Importantly, IMC also proved pharmacological safety in relevant tests [73]. FMC and IMC have demonstrated notable curative effects in lowering plasma TMAO levels and suppressing choline-triggered platelet hyperreactivity and thrombosis, without increasing bleeding risk [85]. Although DMB, IMC, and FMC have shown preliminary efficacy and safety in HF models, clinical data remain scarce. Moreover, Yoshida et al. [28] reported that either administering the FMO inhibitor methimazole or depleting the *FMO2* gene lessened TMAO levels in HF mice, alleviating cardiac fibrosis and dysfunction. Antisense oligonucleotide-targeted inhibition [84] or knockout [144] of the FMO3 gene effectively lowered TMAO levels and subsequently mitigated TMAO-induced platelet hyperreactivity and thrombosis in mice. These results suggest that pharmacological or genetic inhibition of FMOs to decrease TMAO synthesis may offer a therapeutic avenue for HF. Nevertheless, clinical translation constitutes challenges, considering that FMOs participate in the oxidative metabolism of diverse drugs and chemicals in the body besides TMAO. Inhibition or deficiency of FMOs can cause hepatotoxicity and lead to liver diseases [145]. Additionally, loss of FMO3 function in humans may result in harmful TMA buildup, triggering side effects including TMAU [48]. Therefore, potential adverse effects must be thoroughly evaluated before FMO inhibitors are applied clinically.

In conclusion, targeted inhibition of TMAO-producing enzymes represents a promising tactic for addressing HF, whereas significant gaps remain between experimental studies and clinical application, warranting further comprehensive investigation [35].

### 4.5. Modern Medicines

#### 4.5.1. Antibiotics

Antibiotics constitute a conventional strategy for correcting gut microbiota imbalance. They could affect diseases driven by gut microbiota, as they modify its abundance, composition, and metabolic products. Furthermore, HF onset and progression are intimately linked to gut microbial dysbiosis. Research has revealed that antibiotics can ameliorate myocardial ischemia and HF by modulating gut microbiota and its metabolites, suppressing inflammation and oxidative stress, mitigating mitochondrial damage, and bolstering cardiac function [146,147]. In addition, antibiotics decreased TMAO levels by reconstructing gut microbiota, inhibiting the proliferation of harmful gut microbiota, and restricting the conversion of dietary precursors such as choline, L-carnitine, and betaine [35]. In both healthy humans [87] and mice [91], broad-spectrum antibiotics significantly suppressed the dietary phosphatidylcholine-induced increase in plasma TMAO by modulating gut microbiota. Li and coworkers [71] found that antibiotics diminished myocardial hypertrophy and fibrosis in rats by impeding TMAO production. Moreover, depleting gut microbiota with antibiotics attenuated TMAO generation, thereby alleviating ANG II-induced HTN [75]. Broad-spectrum antibiotic therapy also lowered TMAO levels, which decreased macrophage cholesterol content and obstructed the progression of dietary choline-induced AS in mice [91].

However, antibiotic treatment is contentious. Human trials demonstrated that plasma TMAO levels declined markedly during antibiotic administration but rebounded after discontinuation [87]. In a mouse model of MI, antibiotic combination treatment depleted gut microbiota, considerably decreased SCFAs, compromised post-infarction repair, impaired cardiac function, and increased mortality [148]. Additionally, long-term antibiotic use may deplete beneficial gut microbiota and promote antibiotic resistance [149]. Given the numerous detrimental impacts, such as gut microbiota dysbiosis, toxicity, and resistance, a careful risk-benefit assessment is essential before prescribing antibiotics [35]. Further studies are needed to evaluate the potential benefits and safety of rational antibiotic use in patients with HF [93].

#### 4.5.2. Aspirin

Aspirin presents a first-line medication for CVDs and exhibits notable therapeutic impacts on HF with AS [150]. Hazen et al. [151] demonstrated in clinical studies that low-dose aspirin significantly blunted the plasma TMAO elevation caused by a high-choline diet, thereby inhibiting thrombosis and attenuating AS progression. These results were corroborated in a subsequent investigation by Zhu and colleagues [86]. The underlying mechanism may involve aspirin’s capacity to adjust gut microbiota and suppress TMA lyase activity [151,152,153].

#### 4.5.3. Antidiabetic Drugs

Type 2 diabetes mellitus (T2DM) constitutes an independent risk factor for HF, and proactive management of T2DM can effectively reduce the likelihood of developing HF. Metformin, a cornerstone medication for T2DM, confers cardioprotective benefits [154]. Studies confirmed that metformin significantly lowered plasma TMAO concentrations and inhibited TMA generation by gut microbiota in a mouse model of T2DM, independently of choline TMA-lyase activity [155]. Su et al. [156] further discovered that metformin reduced excessive serum TMAO levels in mice fed choline by modifying gut microbial communities involved in choline-TMA conversion. Clinical studies have established the cardiovascular benefits of SGLT2 inhibitors [157], which are now guideline-recommended therapies for HF [158]. Mindrescu et al. [159] observed that empagliflozin altered gut microbiota composition in patients with T2DM, promoting beneficial bacteria such as *Bifidobacterium* and *Lactobacillus* while suppressing proinflammatory genera like *Escherichia* and *Streptococcus*. Experimental studies showed that dapagliflozin attenuated ferroptosis in cardiomyocytes following myocardial ischemia–reperfusion injury in diabetic rats, potentially through gut microbiota modulation and decreased TMAO production [160]. A recent clinical trial found that six-month treatment with either α-glucosidase inhibitor acarbose or the dipeptidyl peptidase-4 inhibitor vildagliptin significantly reduced circulating TMAO levels in overweight or obese patients with T2DM [161]. Nonetheless, no current evidence demonstrates that other antidiabetic agents directly affect TMAO levels.

Therefore, barring any contraindications, antidiabetic drugs could be a viable option to lessen TMAO levels.

#### 4.5.4. Statins

Statins, widely prescribed lipid-lowering agents, exhibit pleiotropic effects such as anti-atherosclerotic, endothelial-improving, anti-inflammatory, antioxidant, antifibrotic, and myocardial energy-enhancing properties [162,163,164]. These multifaceted actions may benefit HF prognosis [94,162,165]. Additionally, Li et al. [166] demonstrated that atorvastatin administration significantly lowered circulating TMAO levels in dyslipidemia patients. Similarly, rosuvastatin therapy in 112 individuals with suspected atherosclerotic cardiovascular disease (ASCVD) improved lipid profiles and notably lessened TMAO concentrations [167].

However, the use of statins in patients with HF remains complex and multifaceted. Although beneficial in many settings, their overall utility in HF patients is not fully established. For instance, different clinical trials in HFpEF populations have not consistently shown significant survival benefits with statin therapy [168,169]. Current guidelines recommend statins for HF patients with concurrent ASCVD, hyperlipidemia, or high cardiovascular risk [2,150]. Patients developing new-onset HF during ongoing statin therapy should maintain treatment unless contraindicated. Clinicians must vigilantly monitor for statin-associated myopathies or hepatotoxicity, necessitating immediate withdrawal upon such adverse events. In hepatic impairment, agents like pravastatin with non-CYP450 metabolism are preferable, whereas high-dose statins should be avoided. Notably, severe liver disease absolutely contraindicates simvastatin, atorvastatin, and lovastatin.

In conclusion, statin use in HF requires careful evaluation of clinical status, HF subtype, and comorbidities. The widespread application of statins in HF still necessitates further research to validate their efficacy and safety. Treatment decisions should therefore be individualized according to patient characteristics and guided by prevailing evidence and guidelines.

#### 4.5.5. ACEIs

ACEIs and angiotensin receptor blockers (ARBs) are highly cost-effective therapies for HF. Studies showed that ACEIs ameliorated host intestinal dysbiosis [170,171]. Konop et al. [172] reported that the ACEI enalapril reduced plasma TMAO levels in rats, suggesting TMAO may serve as a therapeutic target for ACEIs in HF.

Although no direct evidence currently links ARBs to TMAO modulation, these agents are known to influence gut microbiota. Studies showed that ARB treatment reduced blood pressure and alleviated vascular and intestinal injury by altering the diversity, composition, and abundance of gut microbial communities [170,173]. This drug–host–microbiome interaction may extend to angiotensin receptor–neprilysin inhibitors (ARNIs), which incorporate ARB and neprilysin-inhibiting activities. As a foundational HF therapy [174], sacubitril/valsartan belongs to the ARNI class. Research indicated that it ameliorated gut microbiota dysbiosis in murine models of diabetic nephropathy [175].

The above research findings indicate that reducing TMAO levels via modern medicines, including antibiotics, aspirin, antidiabetic drugs, statins, ACEIs, etc., represents a feasible method for managing HF.

### 4.6. Phytomedicines

Phytomedicines can modulate gut microbiota and their metabolites, reduce TMAO concentrations, and exert certain preventive and therapeutic effects on CVDs such as HF [176]. Phytomedicines, distinguished by multi-component, multi-target, and multi-pathway attributes, could alleviate drug resistance via compensatory mechanisms and reduce adverse drug reactions to some extent [177]. They thus provide underlying new strategies for inhibiting TMAO production and treating CVDs.

Research indicated that berberine (BBR), a bioactive compound derived from *Rhizoma coptidis*, showed efficacy in managing HF [178]. BBR reduced TMAO generation in animal models and AS patients by remodeling gut microbiota, downregulating TMA-producing enzymes and FMO3 [179], and suppressing the choline-TMA-TMAO pathway [180], thereby curbing TMAO-induced AS. Importantly, BBR exhibited no hepatorenal toxicity [179]. The findings suggest that BBR possesses significant research and development potential. Resveratrol (RES), a natural extract from plants like *Polygonum cuspidatum*, lowered TMAO levels by decreasing TMA generation via remodeling gut microbiota [181], and was shown to alleviate HF in mice [182]. Garlic (*Allium sativum*), renowned for its antibacterial properties, contains allicin, which has been proven effective in addressing CVDs, including HF [183]. In vitro and in vivo studies confirmed that allicin and fresh garlic juice rich in allicin suppressed the TMAO synthesis through gut microbiota modulation [184]. Recent investigation has revealed that aged garlic oligosaccharides mitigate AS in ApoE^−/−^ mice fed a high-fat and high-cholesterol diet by regulating gut microbiota and decreasing TMAO levels [185]. Hawthorn (*Crataegus*) and its extracts also exhibited therapeutic potential in HF [186,187]. He et al. [92] reported that hawthorn fruit extract lowered TMAO concentrations in mice dose-dependently, mediated by anti-inflammatory and antioxidant properties. Gypenosides (GYP), the primary constituent of *Gynostemma pentaphyllum*, lessened plasma TMAO levels by reshaping gut microbiota and inhibiting TMA lyase activity [188]. Moreover, GYP exerted cardioprotective effects and enhanced cardiac function by promoting mitochondrial autophagy, ultimately ameliorating HF [189]. Ji and coworkers [190] discovered that enemas with rhubarb (*Rhei Radix et Rhizoma*) modulated gut microbiota, decreasing serum TMAO and TMA concentrations and downregulating inflammatory markers in rats. Additional evidence indicated that the main constituents of rhubarb, emodin [191,192] and rhein [193,194], ameliorated pathological cardiac hypertrophy. These findings indicate that rhubarb and its extracts have promise as an innovative therapeutic agent for lowering TMAO concentrations and aiding in managing HF. *Lycium barbarum* polysaccharides (LBPs), important ingredients of *Fructus lycii*, could modulate gut microbiota, reduce intestinal permeability, downregulate inflammatory cytokines, significantly lessen serum TMAO levels, and subsequently improve LV function in mice [195]. Puerarin (PU), derived from *Puerariae lobatae Radix*, enhanced cardiac function in rats suffering from adriamycin-induced HF [196]. A systematic review and meta-analysis showed that combining PU injection with conventional drug therapy was safer and more effective than conventional drug therapy alone in acute heart failure [197]. Recent investigation has demonstrated that PU attenuates TMAO concentrations by altering gut microbiota composition and hindering TMA synthesis, thus exerting anti-AS effects [198]. Polymethoxyflavones (PMFs) from citrus (*Citrus sinensis* L.) peel decreased TMAO levels by inhibiting CutC/D and FMO3 activity, ultimately reducing CVD risk [199,200]. In addition, 5-Demethylnobiletin, a natural PMF, ameliorated isoproterenol-induced cardiac injury in mice [201].

**Figure 3:**
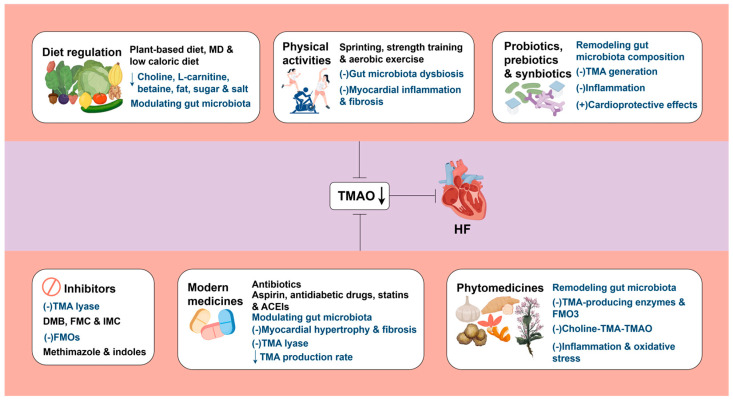
TMAO represents a promising therapeutic target for HF. A downward arrow (↓) indicates a decrease. A plus sign (+) indicates promotion. A minus sign (−) indicates inhibition. MD: Mediterranean diet; TMA: trimethylamine; TMAO: trimethylamine N-oxide; HF: heart failure; DMB: 3,3-dimethyl-1-butanol; FMC: fluoromethylcholine; IMC: iodomethylcholine; FMOs: flavin-containing monooxygenases; ACEIs: Angiotensin-converting enzyme inhibitors. Created by Fidraw (www.figdraw.com, accessed on 10 October 2025).

In summary, TMAO stands out as a potential novel therapeutic target for HF, as illustrated in Figure 3.

## 5. Conclusions and Prospects

HF, a potentially fatal syndrome, poses major obstacles in terms of management, thereby imposing a considerable burden on healthcare systems and affected families. Research has revealed complex relationships between HF pathogenesis and dysregulation of the gut–heart axis. Specifically, patients with HF exhibit gut microbiota dysbiosis with concomitant elevation of the microbial metabolite TMAO. Furthermore, elevated TMAO levels are strongly linked to the occurrence, progression, and prognosis of HF. Therefore, the disparity between TMAO generation and metabolism could constitute a significant mechanism underlying HF. TMAO regulates the advancement of HF via multiple pathways, including inflammation, abnormal energy metabolism, oxidative stress, myocardial remodeling, and so on. Studies have confirmed that decreasing TMAO levels is advantageous in managing HF. Hence, TMAO emerges as a viable target for preventing and treating HF. Several interventions—including healthy diets, regular physical activities, probiotics, prebiotics, synbiotics, inhibitors, antibiotics, aspirin, antidiabetic drugs, statins, ACEIs, and phytomedicines—have shown promising effects in lowering TMAO concentrations and mitigating HF.

However, current research exploring the precise role of TMAO in HF pathogenesis remains limited, particularly in human studies. Many intervention trials often face constraints such as limited sample sizes and insufficient generalizability, necessitating further validation. Furthermore, current research into the mechanisms and treatment of heart failure lacks investigations across distinct phenotypes such as HFpEF and HFrEF, which obscures the phenotype-specific effects of TMAO. Dietary studies have largely been conducted in developed countries, frequently neglecting regional, ethnic, cultural, and customary disparities. Exercise protocols often include sprinting or strength training, which may be unsuitable for HF patients; more research is needed to identify safe and effective activities for those with early-stage HF or at a high risk of developing HF. Clinical trials on other preventive or therapeutic tactics are few, leaving mechanisms and safety in humans incompletely characterized. Interventions using probiotics, prebiotics, and synbiotics encounter limitations, including restricted therapeutic targets and uncertain efficacy, while microbial diversity and environmental complexity complicate the identification of beneficial strains. Although TMA lyase inhibitors have shown preliminary promise, their clinical safety and efficacy require further evaluation. Considering that mutations in the human *FMO* gene may result in conditions including hepatitis and TMAU, utmost prudence is required during the clinical research and development of FMO inhibitors. Antibiotic use is controversial because of potential toxic side effects and the risk of promoting antimicrobial resistance. Therefore, further clinical studies are urgently needed to fully assess the benefits, risks, and prognostic impact of antibiotic therapy. Initial studies have demonstrated that aspirin, antidiabetic drugs, statins, and ACEIs can reduce TMAO levels and HF risk. Phytomedicines possess advantages, including multi-target effects and a lower propensity for drug resistance compared to conventional drugs. Nevertheless, robust clinical data are lacking to fully substantiate the efficacy and underlying mechanisms of these agents. Moreover, the effects of commonly used HF medications—including ARBs, sacubitril/valsartan, and β-adrenergic antagonists—on TMAO levels remain poorly understood.

Furthermore, although cardiac transplantation effectively treats end-stage HF, patients receiving this procedure and its accompanying immunosuppressive therapy often exhibit elevated TMAO levels [202,203]. Several mechanisms may explain this phenomenon. Elevated TMAO precursors were associated with acute rejection and greater atherosclerotic burden [203]. And higher TMAO levels correlated with increased risk of adverse outcomes, including those post-transplantation [97,204], implying that TMAO may already be raised prior to surgery. Immunosuppressed patients are also more vulnerable to infections, and perioperative management often requires broad-spectrum antibiotics. Additionally, heart transplant recipients commonly have multiple comorbidities necessitating polypharmacy. These medications can induce intestinal dysbiosis, which may elevate circulating levels of gut microbiota-derived TMAO [205,206]. Consequently, improved hemodynamics after heart transplantation do not lower TMAO concentrations. In summary, heart transplantation does not mitigate TMAO as a pathological factor in HF. Future studies should further examine the clinical implications of elevated TMAO levels in patients receiving cardiac transplantation and associated immunosuppressive therapy.

Despite these shortcomings and limitations, targeting TMAO remains a promising innovative strategy for preventing and treating HF. More systematic clinical studies are warranted, particularly large-scale, multicenter, randomized controlled trials with sufficient observation durations, clear phenotypes, unambiguous interventions, and precise endpoints. Such efforts would help clarify the mechanisms, efficacy, and safety of TMAO-targeted intervention, providing fresh insights into HF pathogenesis and a scientific foundation for lowering TMAO as a novel strategy in its management.

## Data Availability

Not applicable.

## Abbreviations

The following abbreviations are used in this manuscript:

HF	Heart failure
CVD	Cardiovascular disease
IHD	Ischemic heart disease
LVEF	Left ventricular ejection fraction
HFrpEF	HF with reduced ejection fraction
HFpEF	HF with preserved ejection fraction
AS	Atherosclerosis
SCFAs	Short-chain fatty acids
TMAO	Trimethylamine N-oxide
BAs	Bile acids
LPS	Lipopolysaccharide
TMA	Trimethylamine
FMOs	Flavin-containing monooxygenases
TMAU	Trimethylaminuria
HFD	High-fat diet
IL	Interleukin
NLRP3	Nucleotide-binding oligomerization domain-like receptor containing pyrin domain 3
SIRT3	Sirtuin 3
SOD2	Superoxide dismutase 2
mtROS	Mitochondrial reactive oxygen species
ROS	Reactive oxygen species
TXNIP	Thioredoxin-interacting protein
TNF	Tumor necrosis factor
FA	Fatty acid
OXPHOS	Oxidative phosphorylation
PLIN4	Perilipin 4
OMA1	Overlapping activity with m-AAA protease 1
OGDHL	Oxoglutarate dehydrogenase L
BAT	Brown adipose tissue
CP	Creatine phosphate
ATP	Adenosine triphosphate
Ca^2+^	Calcium ion
eNOS	Endothelial nitric oxide synthase
NOX2	NADPH oxidase 2
PIEZO1	Piezo type mechanosensitive ion channel component 1
WD	Western diet
ANP	atrial natriuretic peptide
β-MHC	β-myosin heavy chain
TGF	Transforming growth factor
NF	Nuclear factor
BNP	Brain natriuretic peptide
LV	Left ventricular
NO	Nitric oxide
CHF	Chronic heart failure
HTN	Hypertension
ANG	Angiotensin
MI	Myocardial infarction
RCT	Reverse cholesterol transport
ApoE^−/−^	Apolipoprotein E knockout
CADs	Coronary artery diseases
SGLT2	Sodium-glucose cotransporter-2
ACEIs	Angiotensin-converting enzyme inhibitors
MD	Mediterranean diet
MF	Multistrain formula
DMB	3,3-dimethyl-1-butanol
FMC	Fluoromethylcholine
IMC	Iodomethylcholine
T2DM	Type 2 diabetes mellitus
ASCVD	Atherosclerotic cardiovascular disease
ARBs	Angiotensin receptor blockers
ARNIs	Angiotensin receptor–neprilysin inhibitors
BBR	Berberine
RES	Resveratrol
GYP	Gypenosides
LBPs	Lycium barbarum polysaccharides
PU	Puerarin
PMFs	Polymethoxyflavones
